# Spatial Analysis of Human Health Risk Due to Arsenic Exposure through Drinking Groundwater in Taiwan’s Pingtung Plain

**DOI:** 10.3390/ijerph14010081

**Published:** 2017-01-14

**Authors:** Ching-Ping Liang, Yi-Chi Chien, Cheng-Shin Jang, Ching-Fang Chen, Jui-Sheng Chen

**Affiliations:** 1 Department of Nursing, Fooyin University, Kaohsiung 831, Taiwan; sc048@fy.edu.tw; 2 Department of Environmental Engineering and Science, Fooyin University, Kaohsiung 831, Taiwan; pl036@fy.edu.tw; 3 Department of Leisure and Recreation Management, Kainan University, Taoyuan 338, Taiwan; csjang@mail.knu.edu.tw; 4 Graduate Institute of Applied Geology, National Central University, Taoyuan 320, Taiwan; fafa7250@yahoo.com.tw

**Keywords:** health risk, arsenic, spatial analysis, hazard quotient, target risk, Kriging method

## Abstract

Chronic arsenic (As) exposure continues to be a public health problem of major concern worldwide, affecting hundreds of millions of people. A long-term groundwater quality survey has revealed that 20% of the groundwater in southern Taiwan’s Pingtung Plain is clearly contaminated with a measured As concentration in excess of the maximum level of 10 µg/L recommended by the World Health Organization. The situation is further complicated by the fact that more than half of the inhabitants in this area continue to use groundwater for drinking. Efforts to assess the health risk associated with the ingestion of As from the contaminated drinking water are required in order to determine the priorities for health risk management. The conventional approach to conducting a human health risk assessment may be insufficient for this purpose, so this study adopts a geostatistical Kriging method to perform a spatial analysis of the health risk associated with ingesting As through drinking groundwater in the Pingtung Plain. The health risk is assessed based on the hazard quotient (HQ) and target cancer risk (TR) established by the U.S. Environmental Protection Agency. The results show that most areas where the HQ exceeds 1 are in the southwestern part of the study area. In addition, the high-population density townships of Daliao, Linyuan, Donggang, Linbian, Jiadong, and Fangliao presently have exceedingly high TR values that are two orders of magnitude higher than the acceptable standard. Thus, the use of groundwater for drinking in these townships should be strictly avoided. A map that delineates areas with high TR values and high population densities is provided. The findings broaden the scope of the spatial analysis of human health risk and provide a basis for improving the decision-making process.

## 1. Introduction

Arsenic (As) whether from natural sources or anthropogenic activities is widely distributed in the subsurface environment, but elevated levels of As in the groundwater occur most notably in parts of Bangladesh [1], West Bengal [2], the United States [3,4], and Taiwan [5]. Arsenic in the groundwater is considered a serious problem because exposure is known to cause a variety of acute and chronic human health problems. Epidemiological studies show that exposure to inorganic As is found to increase the risk of cancer. Thus, the International Agency for Research on Cancer (IARC) classifies As and As compounds as Group 1 carcinogens. The U.S. Environmental Protection Agency (U.S. EPA) also lists aresenic as a Group A, or known carcinogen. The carcinogenic effects of arsenic in the drinking water have been reported globally [6]. Increased mortality from various cancers has also been observed among residents in the endemic southwestern area compared with the general population in Taiwan [7]. There is a significant association between an in the elevation in the mortality rate due to cancer and the use of artesian well water for drinking, showing a dose-response relation [7,8]. Intensive analysis of the risk of cancers of the liver, lung, and urinary tract associated with arsenic in the drinking water in southwestern Taiwan has shown that [9] although both genetic and acquired individual susceptibility may modify the carcinogenic effect of arsenic on the human body [10,11], arsenic remains an independent predictor of the risk of related cancers, even after taking other factors into consideration.

The main route of As exposure for the general population is the ingestion of As through drinking water and food [12,13,14]. The U.S. EPA has specified the standard for acceptable levels of As in public drinking water at a maximum contaminant level (MCL) of 10 μg/L [15]. This is also the regulatory standard for drinking water in Taiwan. Past studies show that elevated levels of As were found in groundwater in areas with blackfoot disease (BFD) in southwestern Taiwan during the 1960s [5]. The correlation between As contamination and chronic As-related health problems has been well documented. In addition to the historical BFD region, high As levels have also been found in groundwater in several other regions of Taiwan, including Ilan, Yunlin, Chiayi, Tainan, and Pingtung counties. Nowadays, most local residents in southern Taiwan consume tap water processed by the public water company, but a large amount of As-contaminated groundwater is still used for aquaculture. As can accumulate in the tissue of farmed fish and shellfish and recent studies have been carried out to evaluate exposure to As through the consumption of seafood in Taiwan and the related health risk assessment [16,17,18,19,20,21,22,23,24,25].

Although the consumption of seafood has become a major route for people in Taiwan to be exposed to As, drinking As-contaminated groundwater remains the major means of exposure. Lee et al. [26] evaluated the potential health risks of drinking groundwater containing As for the residents of the Lanyang Plain in northeastern Taiwan. The residents of the Pingtung Plain, which is located in southern Taiwan, use a substantial amount of groundwater which is relatively abundant and inexpensive to meet their drinking, agriculture and aquaculture requirements. According to statistics from the Taiwan Water Resources Agency (WRA), only 46.89% of the population in the Pingtung Plain has piped in tap water, far below the national average of 92.93%. In other words, groundwater is still in widespread use for household purposes and for drinking. Long-term groundwater quality monitoring data indicate that the As content in groundwater in the Pingtung Plain exceeds the Taiwan Environmental Protection Agency (EPA)’s drinking water quality standard of 10 μg/L, by over 20%. Understanding the potential threat due to As intake from the groundwater is a critical environmental and public health concern. Recently, Liang et al. [27] evaluated the exposure and health risk from drinking groundwater for residents in the Pingtung Plain. However, their estimate did not account for spatial variability of the arsenic concentration in the groundwater.

The spatial distribution of As pollutants in the groundwater is usually heterogeneous and can vary significantly from region to region. The implication is that the human health risk may also vary from region to region corresponding to variations in the level of arsenic in the groundwater and the amount used as the source of drinking water. This spatial variability has been neglected in the conventional approach to conducting a health risk assessment. Past methods are insufficient for effective management of the actual health risk to the population of the Pingtung Plain. Clearly, there is an urgent need to develop an advanced health risk assessment method that takes into account variability. This study performs a spatial analysis of the health risk associated with As exposure through the drinking of groundwater in Pingtung Plain. The results are expected to indicate specific regions where the health risk is high which would help authorities to develop more effective health management plans.

## 2. Materials and Methods

A general framework is presented herein for spatial analysis of the health risk of arsenic intake from the drinking of groundwater in the Pingtung Plain. The non-carcinogenic hazard quotient (HQ) and carcinogenic target cancer risk (TR) models recommended by the U.S. EPA are used to perform the health risk assessment. First, the spatial distributions of the As concentrations are calculated using the Kriging geostatistical approach. Subsequently, the spatial patterns of the HQ and TR are computed based on the spatial distribution of the As concentrations. Integrating the mapping of the TR values in individual townships with the population densities of these townships can help water management agencies prioritize the areas where an effective health management plan is imperative to reduce the intake of As and to supply residents with safe drinking water.

### 2.1. Study Area and Hydrogeology

**Figure 1:**
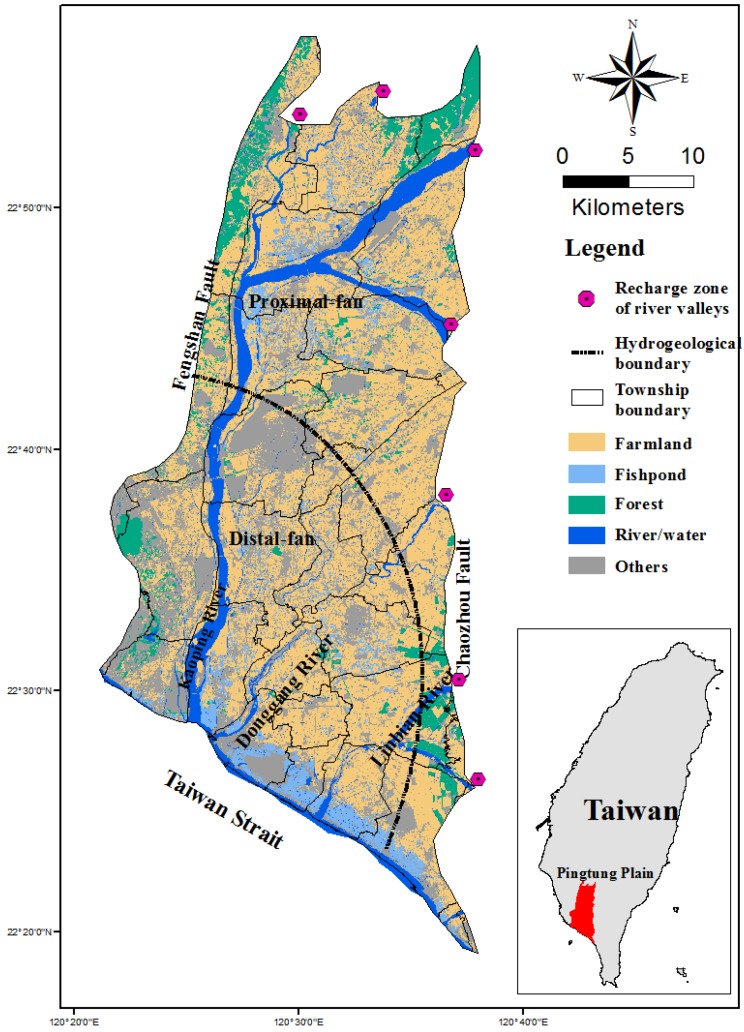
Land use in the study area.

**Figure 2:**
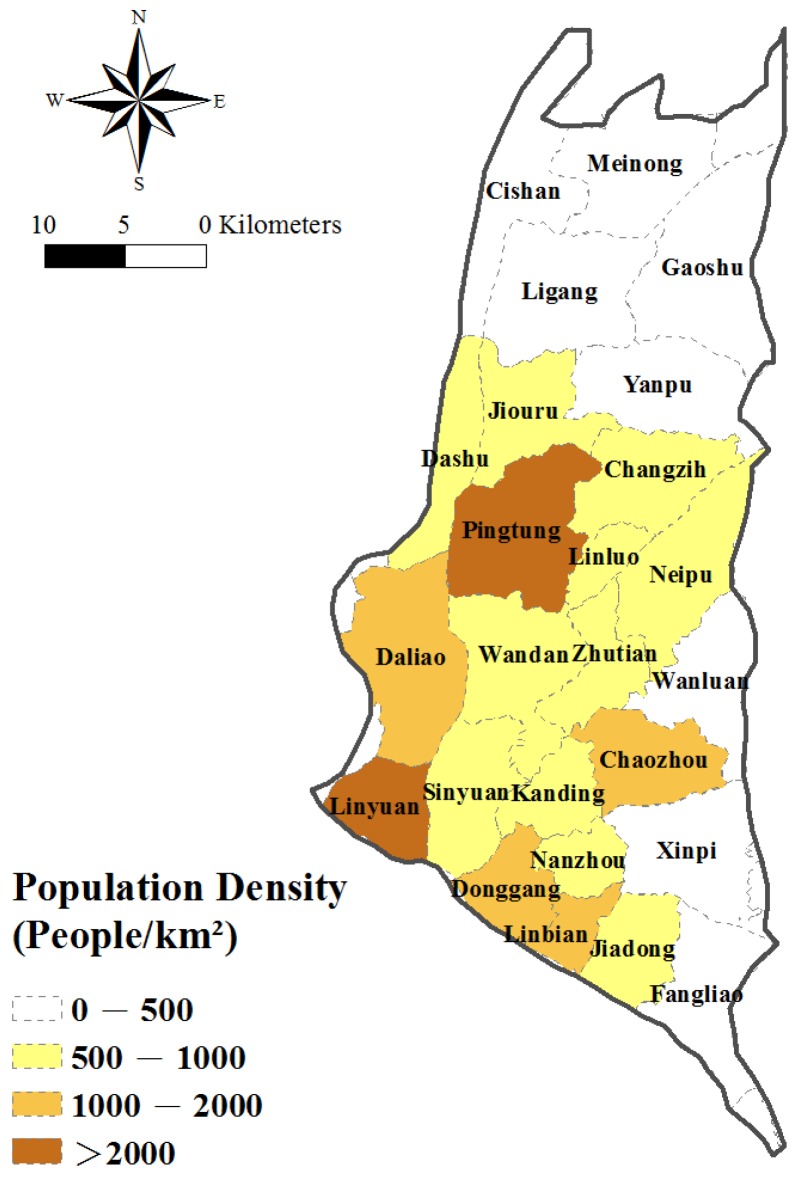
Population densities of townships in the study area.

The Pingtung Plain is situated in southern Taiwan and has a total area of 1210 km^2^ (Figure 1). It comprises 30 townships and has a population of more than 870,000, but the population density has a non-uniform distribution, as shown in Figure 2. The area includes the alluvial fan of the Kaoping River, which has the largest drainage area of all rivers in Taiwan. Other shorter rivers such as the Tungkang River, Linbian River and Shihwen River also pass through the plain which faces the Taiwan Strait on the southwest, to the east of Kaohsiung City, and is bound on the west by the Central Mountain Range.

**Figure 3:**
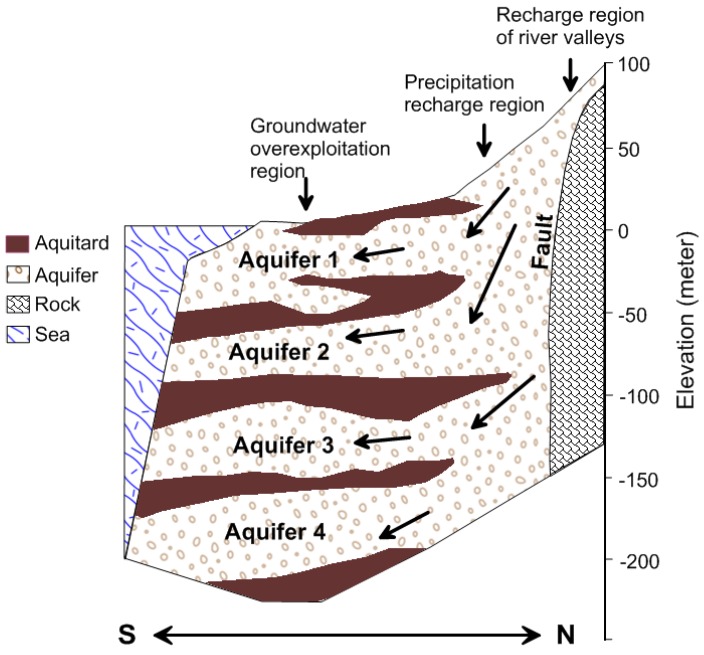
Hydrogeological profile of the study area.

The geology underlying the plain primarily consists of unconsolidated sediments from the Late Pleistocene and the Holocene age and contains abundant groundwater. Several drilling studies and stratigraphic analyses of the subsurface geology and hydrogeology were conducted from 1995 to 1998. The subsurface hydrogeological analysis was completed to a depth of approximately 250 m and the results show the plain to be partitioned primarily into proximal-fan and distal-fan areas. The deposits in the distal fan area can be grouped into eight overlapping sequences, including four marine sequences and four non-marine sequences [28]. The non-marine sequences, comprised of highly permeable coarse sediment are considered to be aquifers, whereas the marine sequences, comprised of less permeable fine sediment are regarded as aquitards [29] (Figure 3). The aquitards are found mainly in the distal-fan area. They are not present in the proximal-fan area. Four usable aquifers, can be seen in this figures, labeled Aquifer 1, Aquifer 2, Aquifer 3 and Aquifer 4, from top to bottom, at depths of 0–70 m, 40–130 m, 90–180 m, and 160–250 m, respectively.

The principal source of freshwater in the plain originates from the infiltration of natural rain into the groundwater which collects in the principal, ancient Quaternary reservoir and is extracted by wells. The proximal-fan area and the river valleys on the eastern and northern boundaries are the major regions for aquifer recharging. Groundwater flows from these areas toward the southwestern areas bordering the Taiwan Strait. The period of maximum precipitation in the Pingtung Plain is generally from May to September (with an average accumulation of 2493 mm per year), followed by considerably lower precipitation from October to December and January to April. The seasonality of the precipitation has resulted in a reliance on irrigation for the cultivation of crops and for aquaculture involving the legal and sometimes illegal extraction of surface water and groundwater.

The bulk of the water required for use in the highly-productive agricultural areas of the Pingtung Plain is supplied by groundwater. Figure 1 presents a map of land use. It can be seen that approximately 45.7% of the area is used for agriculture, and 5.1% for fishponds. Over the past decade agricultural activities have continued to intensify. In the dry months and years, large amounts of groundwater are extracted to meet the water resource requirements for farmlands, fishponds and households. This has led to an increase in the salinity of the groundwater, a reduction in the pollution diluting capability of the surface water, and an increase in the occurrence of severe land subsidence and seawater intrusion [30].

### 2.2. Groundwater Samples

A project for characterizing the subsurface hydrogeology of the Pingtung Plain and observing the long-term groundwater level was conducted by Taiwan’s Water Resource Agency (WRA) from 1995 to 1998. For this project a groundwater observation network with 51 boreholes drilled and 126 wells screened in different aquifers with various depths was established. With financial support from the Taiwan WRA, the Agricultural Engineering Research Center (AERC) periodically conducts groundwater quality surveys. The AERC groundwater quality survey includes 31 items including As [31,32,33,34] and other potentially hazardous chemicals. Arsenic concentrations were analyzed based on the APHA Method 3500-AsB. The method detecting limit was 0.1 μg/L.

### 2.3. Geostatistical Approach

The spatial distributions of the As concentrations in the study area are estimated using the geostatistical Kriging approach, the core of which is the regionalized variable theory, which states that variables in an area exhibit both random and spatially structured properties [35]. In the geostatistical approach the regionalized variable is typically assumed to be second-order stationary. A geostatistical variogram of the data within a statistical framework needs to be determined first. The variogram is used to measure the spatial variability of the random variables between two locations.

The semi-variogram, γ(h), is defined as follows:

(1) $$\gamma (h)=\frac{1}{2N(h)}\left\{\sum_{i=1}^{N(h)}{\left[Z(x_{i}+h)-Z(x_{i})\right]}^{2}\right\}$$

where *h* denotes the lag; *Z*(*x_i_*) is the value of the regional variable of interest at location *x_i_*, *Z*(*x_i_* + *h*) is the value of the regional variable of interest at location *x_i_* + *h*; and *N*(*h*) is the number of pairs of sampling points separated by *h*. In practice, the probability of the distance between the sampled pairs being exact is low, thus *h* is represented by a distance interval.

The experimental semi-variogram of the sampling data is fitted against a theoretical semi-variogram model of *γ*(*h*). The widely used theoretical spherical, exponential and Gaussian models can be written as follows:

(2) $$\gamma (h)=\{\begin{array}{c} c_{0}+c\left[1.5\left(\frac{h}{a}\right)-0.5{\left(\frac{h}{a}\right)}^{3}\right]\;h\leq a\\ c_{0}+c\;h>a \end{array}\text{ spherical model};$$

(3) $$\gamma (h)=c_{0}+c\left[1-\exp\left(-\frac{3h}{a}\right)\right]\text{ exponential model};$$

(4) $$\gamma (h)=c_{0}+c\left\{1-\exp\left[-{\left(\frac{3h}{a}\right)}^{2}\right]\right\}\text{ Gaussian model};$$

where *c*_0_ is the nugget effect; *c* is the sill and *a* is the range.

The theoretical semi-variogram models provide information about the spatial structure and input parameters required for geostatistical Kriging interpolation. The Kriging method is regarded as an optimal spatial interpolation method in which the values of the random field at an unsampled location *x*_0_ are estimated on the basis of the linear combination of the given values of the measured locations as follows:

(5) $$Z*(x_{0})=\sum_{i=1}^{i=M}\lambda_{i0}Z(x_{i})$$

where $Z*(x_{0})$ is the value at an unsampled location to be estimated at *x*_0_; *Z*(*x_i_*) denotes the given value at a sampled location (*x_i_*); *M* is the total number of given sampled values used for estimation; and *λ_i_*_0_ is the Kriging weight for *Z*(*x_i_*) which is used to estimate $Z*(x_{0})$.

### 2.4. Human Health Risk Assessment

The purpose of the health risk assessment is to estimate to what extent the population’s health would be at risk through drinking As contaminated water. The health risk from both non-carcinogenic and carcinogenic exposure arising from the intake of As is considered. The health risk for such exposure is estimated using the method recommended by the U.S. EPA.

The health risk for non-carcinogenic exposure is evaluated based on the hazard quotient (HQ) index which is defined as the ratio of the potential exposure to a level at which no adverse effects are expected. An adverse non-carcinogenic effect is regarded as possible if the calculated HQ value is greater than 1; less than 1, then no non-carcinogenic effects are expected. The hazard quotient (HQ) is calculated by:

(6) $$HQ=\frac{DI}{RfD}$$

where *DI* is the daily intake of As (mg/kg body weight/day) and *RfD* is the oral reference dose derived by the U.S. EPA [36].

Daily intake in Equation (6) is calculated based on the widely used model derived by the U.S. EPA [37] as follows:

(7) $$DI=\frac{C_{w}\times IR}{B_{w}}$$

where *C_w_* is the As concentration in the groundwater (mg/L); *IR* is the daily water intake rate of an adult (L/day); and *B_w_* is body weight (kg).

Health risk for carcinogenic exposure is evaluated based on the target cancer risk (TR) index which is expressed as the excess probability of contracting cancer over a lifetime of 70 years. Generally, the health risk for carcinogenic exposure is acceptable if the TR is lower than the threshold value of 10^−6^. The model for estimating target cancer risk (lifetime cancer risk) is formulated as:

(8) $$TR=\frac{C_{w}\times IR\times EF\times ED\times CSF}{BW\times AT}\times 10^{-3}$$

where *EF* is the exposure frequency (day/year); *ED* is the exposure duration (year); *CS*F is the cancer slope factor (per mg/kg/day) obtained from the Integrated Risk Information System (IRIS) database (1.5/(mg/kg/day)); and *AT* is the average time for carcinogenic exposure (25,550 days). In Equation (8), 10^−3^ is a conversion factor. The exposure duration is defined as the frequency of exposure for 365 days/year over 30 years (i.e., *EF* × *ED* = 10,950 days). Table 1 shows the parameters used for target cancer risk estimation.

## 3. Results and Discussion

### 3.1. Spatial Distribution of Arsenic Concentrations

First, we perform a descriptive statistical analysis of the arsenic concentration data for the Pingtung Plain collected from the Taiwan’s Water Resources Agency from 2009 to 2013. The statistics for the As concentrations measurement at the monitoring wells are summarized in Table 2. There is a considerable variation in the measured concentrations, from below the detection limit (<0.1 µg/L) to the maximum value of 544 µg/L. The maximum arsenic concentration is 50 times the threshold value of 10 µg/L recommended by the WHO. The average As concentration is 18.1 μg/L with a standard deviation of 65.2 μg/L. Moreover, the threshold value of 10 µg/L corresponds to the 80.26th percentile of the percentage frequency distribution of arsenic concentration. In other words, approximately 20% of the measured As concentrations exceeds the threshold value of 10 μg/L.

**Figure 4:**
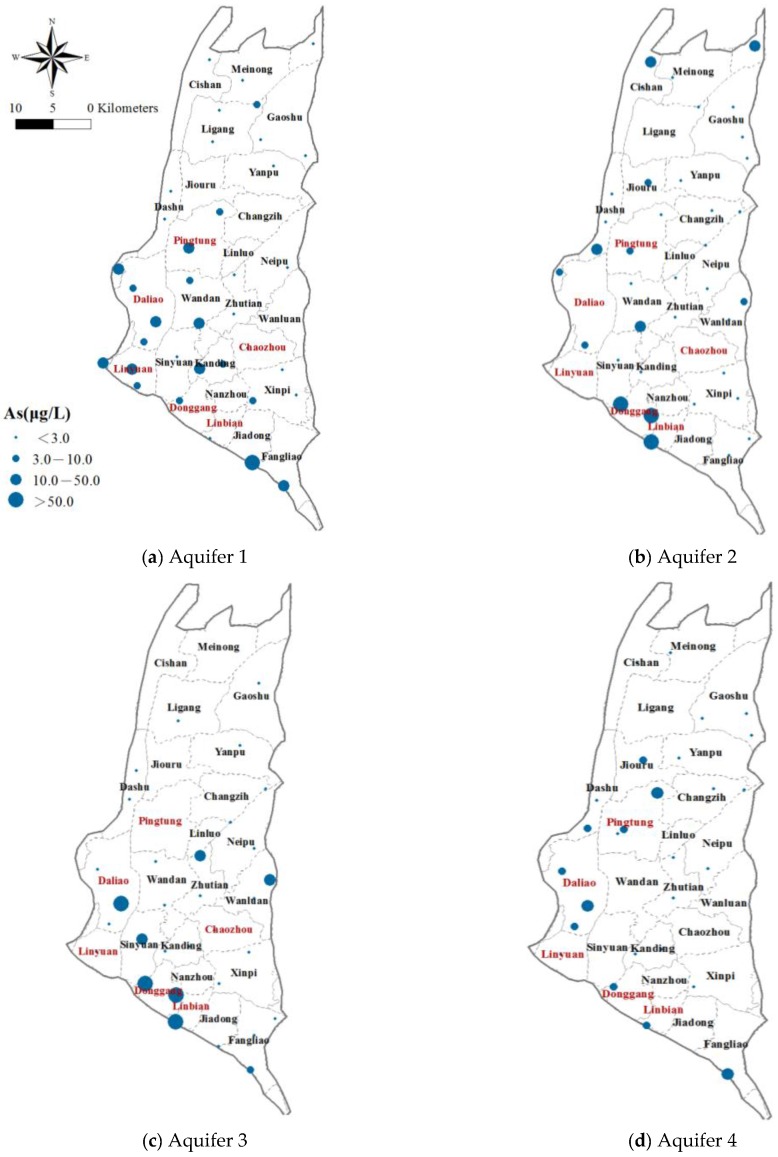
Measured arsenic concentration at the monitoring wells established by Taiwan’s WRA.

Efforts are made to clarify the spatial distribution of arsenic concentrations in the groundwater; the measured concentrations in each aquifer are depicted in Figure 4. The measured As concentrations are categorized into four levels based on Taiwan’s drinking water quality standard: completely uncontaminated (<3 μg/L), moderately uncontaminated (3–10 μg/L), moderately contaminated (10–50 μg/L), and severely contaminated (>50 μg/L). In the figure, the As levels are represented by the sizes of the solid circles. There is a pattern with obvious increase in the As concentration in Aquifers 1, 2 and 3 from the northeastern to the southwestern coastal area with most of the groundwater samples exceeding 10 μg/L located in the southwestern part of the study area. A few others are scattered in the central and northern areas. In Aquifer 4, in the western and southern part of the study area the arsenic concentrations are high.

Next, the spatial pattern of the arsenic concentrations is analyzed and interpolated into a GIS environment using the geostatistical Kriging method. A histogram of the arsenic concentrations is prepared prior to calculating the semi-variogram, reveals a lognormal distribution rather than a normal distribution. Thus, log transformation of the measured arsenic concentrations is used for calculation of the semi-variogram. Comparison of the calculated semi-variograms for the logarithms of arsenic concentrations with different theoretical semi-variogram models shows that the Gaussian model has the best fit.

**Figure 5:**
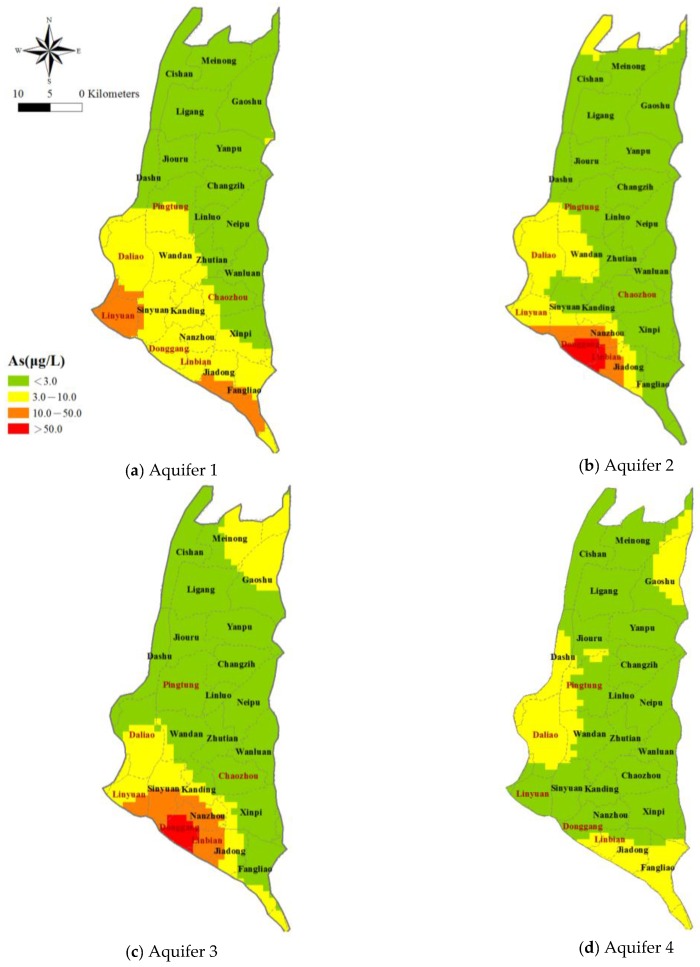
Spatial distribution of arsenic concentrations mapped using the geostatistical method.

The theoretical Gaussian semi-variogram model is selected and the spatial distribution of the arsenic concentration is estimated by calculating the exponent transformation of the estimated values of the logarithmic concentrations for each cell using Equation (5). Each aquifer is discretized into a grid system of 2448 cells with a cell size of 1000 m × 1000 m. Figure 5 presents the spatial distribution of the estimated arsenic concentration obtained using the geostatistical Kriging method. The arsenic concentrations in Aquifer 1 are higher in the townships of Linyuan, Linbian, Sinyuan and Jiadong. In Aquifer 2, the arsenic concentrations are higher in the townships of Dongang, Linbian and Nanzhou. For Aquifer 3, the areas with higher arsenic concentrations cover the townships of Dongang, Linbian and Nanzhou. Only a small portion of the area associated with Aquifer 4 has an excessive arsenic concentration. Overall, the highest As content is apparent in Aquifers 2 and 3 in the southwestern area. The lower As concentrations in Aquifer 4 indicate that this may be a suitable safe zone for the withdrawal of groundwater. The As concentrations are obviously lower in the northern and eastern parts of the study area, in Aquifers 1–4, indicating that they also could be primary safe water sources.

### 3.2. Spatial Arsenic Risk Assessment and Health Risk Implications

**Figure 6:**
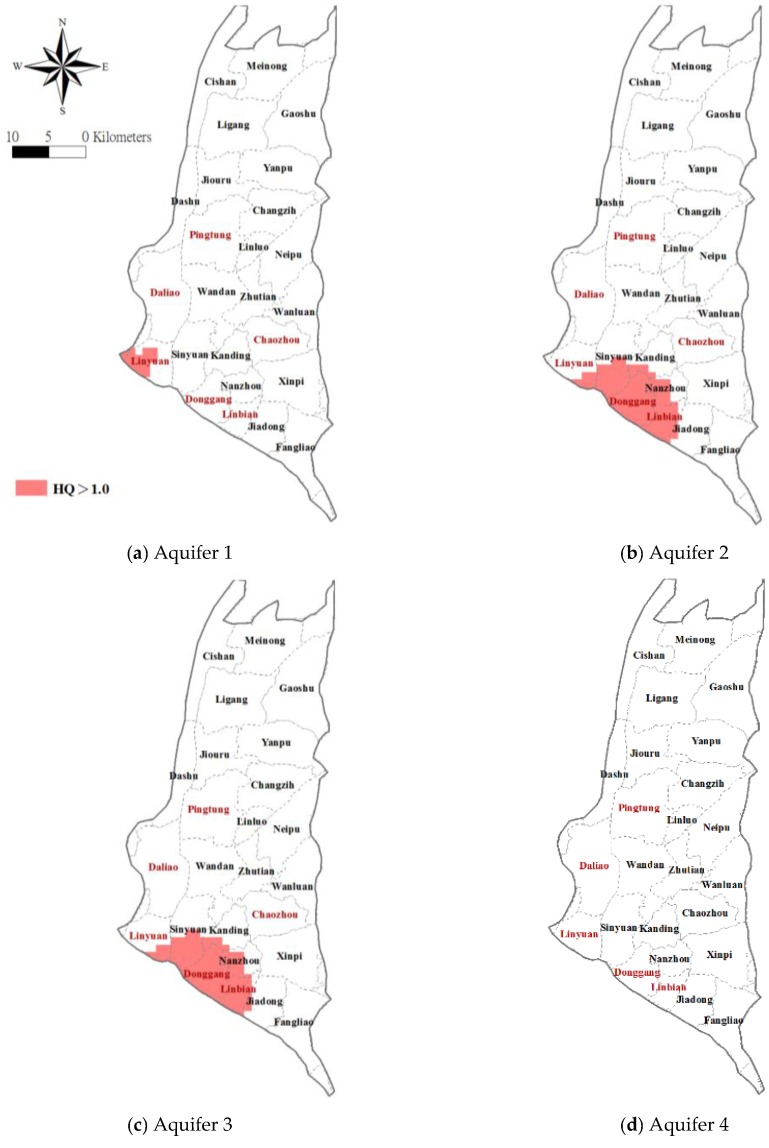
Spatial distribution of estimated hazard quotient (HQ) with HQ values >1 indicated in red.

Given the known health risks associated with the ingestion of As, we set out to identify the areas of concern and quantitatively assess the health risk of drinking As-contaminated groundwater throughout the Pingtung Plain. The spatial distribution of the hazard quotient (HQ) index for non-carcinogenic health risk is evaluated using Equation (6) with the aid of estimated arsenic concentrations in each cell obtained from the geostatistica Kriging approach. Figure 6 maps the estimated spatial distribution of HQ values corresponding to each aquifer associated. The cells where HQs > 1 are indicated in red and are located primarily in the southwestern part of the study area, especially in the townships of Linyuan for Aquifer 1, Sinyuan, Dongdang, Linbian and Nanzhou for Aquifers 2 and 3. The estimated HQs for Aquifer 4 are all less than the 1.0 throughout the entire study area.

**Figure 7:**
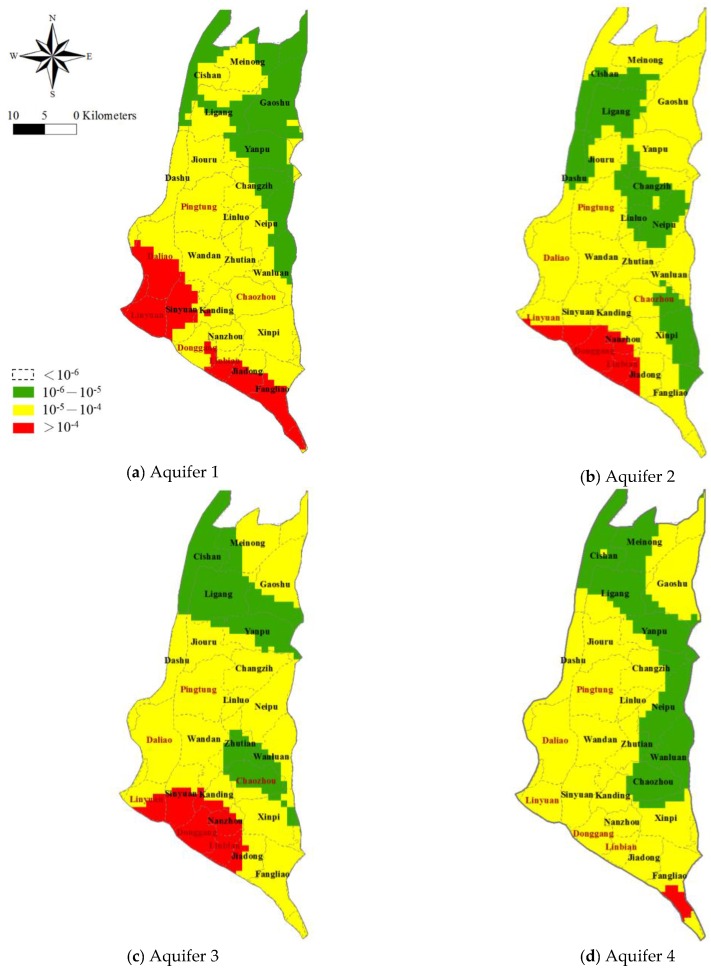
Spatial distribution of estimated target cancer risk (TR). The TR values are classified as <10^−6^, 10^−6^–10^−5^, 10^−5^–10^−4^ and >10^−4^.

Taking the estimated arsenic concentrations for each cell for each aquifer from the previous section, the spatial distribution of the target cancer risk (TR) index is evaluated using Equation (8). Figure 7 maps the estimated TRs for each aquifer. The estimated TR values are 1.8 to 1890 times higher than the acceptable standard (one millionth, 10^−6^) throughout the study area, for each aquifer. In Figure 7, the areas with the highest TR values (>10^−4^) are indicated in red. For Aquifer 1, the TR values are highest in the townships of Daliao, Linyuan, Sinyuan, Donggang, Linbian, Jiadong and Fangliao (>10^−4^). For Aquifers 2 and 3, the areas with the exceedingly high TRs include the townships of Linyuan, Sinyuan, Donggang, Linbian and Jiadong (JD). In Aquifer 4, most areas display TRs ranging from 10^−5^ to 10^−4^ with the exception of a small portion of the township of Fangliao which has TR values greater than 10^−4^. It should be noted that most of the townships with high TRs have larger population densities. The areas with high TRs and high population densities appear to be facing a more severe public health issue and thus deserve special attention.

The percentage the area where the TR values are estimated to be in excess of 10^−5^ and 10^−4^ and the population densities for the individual townships in the Pingtung Plain are shown in Table 3. Examination of the results show that an exceedingly high percentage of the population is exposed to high carcinogenic risk from As-affected groundwater. Most of the high population density townships have a level of risk exceeding 10^−5^.

**Figure 8:**
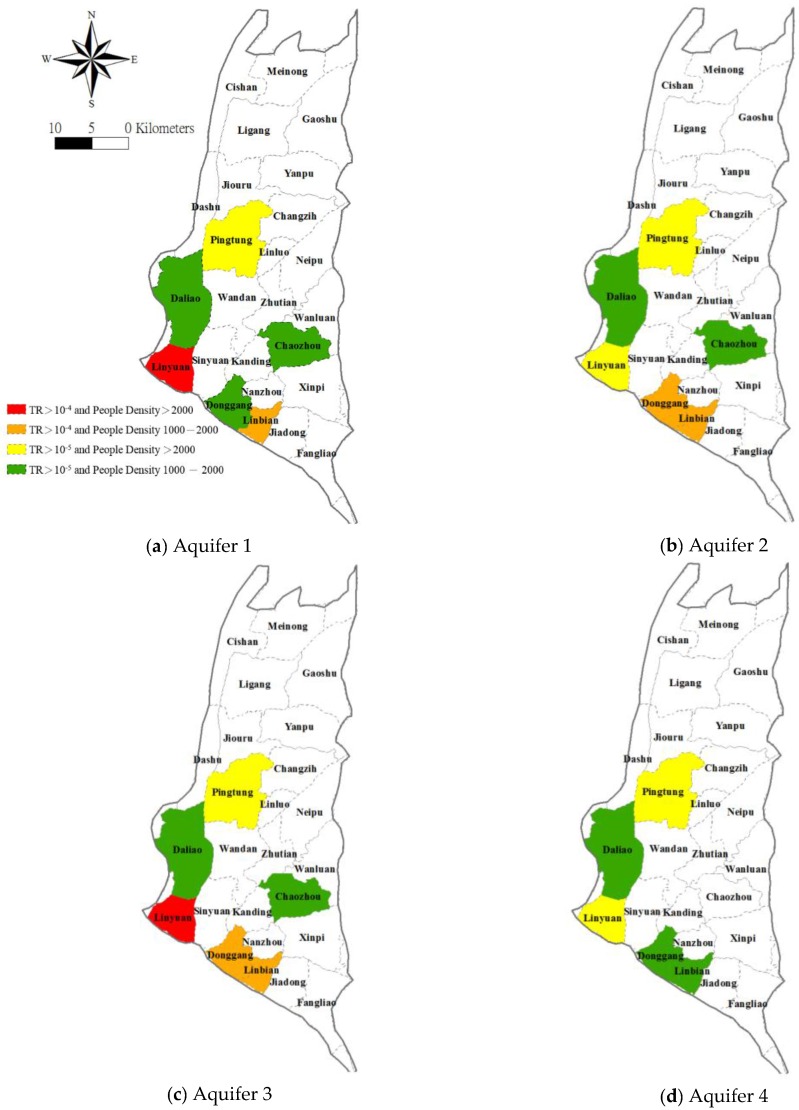
Zonal risk calculated based on target cancer risk (TR) and population density.

Given the area percentage (%) where the estimated TR exceeds 10^−5^ and the actual population of individual townships, it is simple to estimate that more than the health of 83.9%, 78.7%, 81.3% and 75.9% of the population corresponding Aquifers 1, 2, 3, and 4, respectively, is at risk due to the ingestion of As containing groundwater. Furthermore, over 17.1%, 10.7%, 13.3% and 0.4% (for Aquifers 1, 2, 3 and 4 respectively) of the population have been exposed to a level of risk exceeding 10^−4^. A spatially explicit map that delineates the high population density areas where residents are exposed to greater carcinogenic risk can be prepared by integrating the spatial distribution of the estimated TR and population density; see the map in Figure 8 based on TR > 10^−4^ and population density (PD) > 2000 person/km^2^. Four classes of exposure are considered.

**Figure 9:**
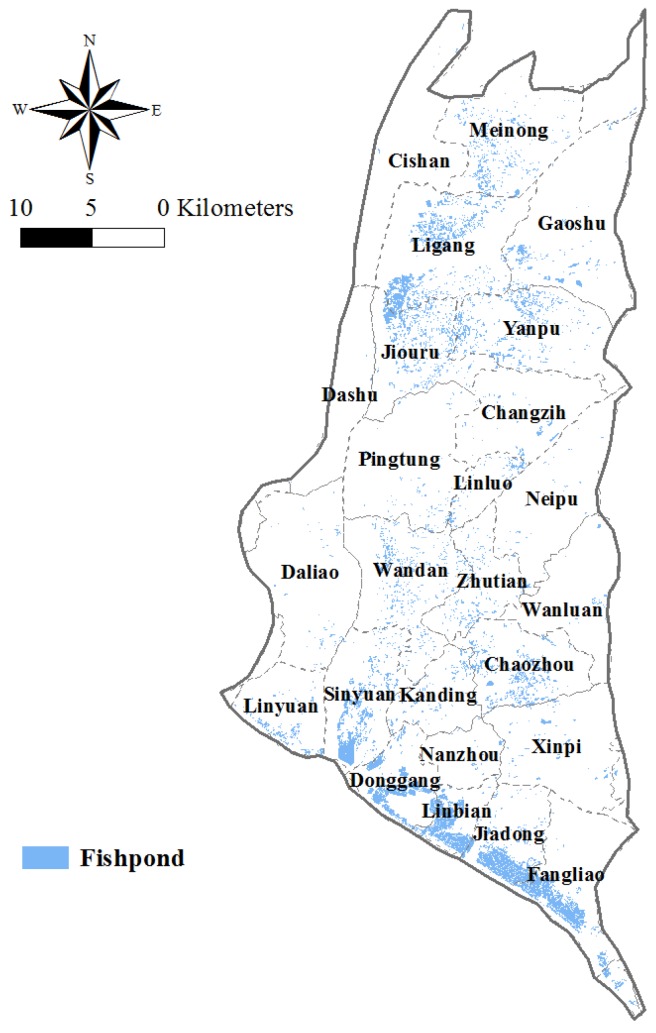
Fishpond locations in the study area.

In addition to the ingestion of As from drinking groundwater, the consumption of the contaminated foodstuff is also an important exposure source. Contaminated groundwater is also being used for aquaculture. The accumulation of As in farmed fish and seafood pose a potential threat to human health [19,21,22,23]. Figure 9 shows fishpond locations in the study area. Many are located in the southwestern part of the Pingtung Plain and in the higher population density townships, which are exactly in the higher risk areas as estimated in our study. The exposure associated with the consumption of fish and seafood actually increases the carcinogenic risk and should be considered in the future.

## 4. Conclusions

A long-term groundwater quality survey has revealed that 20% of the measured As concentrations in groundwater in the Pingtung Plain, in southern Taiwan, clearly reach or exceed the level of 10 µg/L recommended by the WHO. The situation is further complicated because more than half of the inhabitants of the Pingtung Plain still use groundwater for drinking. Efforts to assess the health risk associated with the consumption of As through contaminated drinking water should be required to help define priorities for health risk management. This study uses the geostatistical Kriging method to perform spatial analysis to map the health risk associated with ingesting As through drinking groundwater in the Pingtung Plain. The spatial distribution of the hazard quotient (HQ) and target cancer risk (TR) indexes are mapped. The results show that most areas with HQs exceeding 1 are found in the southwestern part of the study area. It is also found that the high-population density townships of Daliao, Linyuan, Donggang, Linbian, Jiadong and Fangliao have exceedingly high TR values, two orders of magnitude higher than the acceptable standard. The areas with high TR values and high population densities are mapped. The results are expected to improve the decision-making process. It is imperative that the government adopt effective measures to ensure the supply of safe drinking water, especially for those township with high TR values and high population densities.

## Figures and Tables

**Table 1:** Parameters used in the health risk model.

Parameter (Unit)	Parameter Characteristics
ED (year)	30
EF (day)	365
IR (L/day)	1.43
BW (kg)	64.6
AT (day)	79.0 × 365 = 28,835
RfD (mg/kg/day)	3 × 10^4^ [35]
CSF (mg/kg/day)^−1^	1.50 [35]
C (μg/kg)	From groundwater quality monitoring data of Taiwan WRA

**Table 2:** Statistics regarding measured concentrations of As at the monitoring wells (µg/L).

Statistics	As Concentration (µg/L)
Well number	132
Average	18.1
Median	0.9
Std. Deviation	65.2
Skewness	5.8
Minimum	<0.1
Maximum	544.0
Percentiles	
50th	0.9
60th	2.1
70th	4.6
80th	9.6
80.26th	10.0
90th	24.8

**Table 3:** Area percentage (%) of target cancer risk exceeding 10^−5^ and 10^−4^ in individual townships on the Pingtung Plain.

Township	Population Density	Population	Area Percentage of TRs > 10^−5^				Area Percentage of TRs > 10^−4^			
	(Person/km^2^)	(Person)	F1	F2	F3	F4	F1	F2	F3	F4
Pingtong	3133.17	203,866	100.0%	87.4%	100.0%	100.0%	0.0%	0.0%	0.0%	0.0%
Linyuan	2182.87	70,476	100.0%	100.0%	100.0%	100.0%	100.0%	34.0%	42.6%	0.0%
Donggang	1638.03	48,262	100.0%	100.0%	100.0%	100.0%	13.6%	93.2%	100.0%	0.0%
Daliao	1565.19	111,191	85.7%	85.7%	85.7%	85.7%	34.7%	0.0%	0.0%	0.0%
Chaozhou	1289.98	54,738	100.0%	71.7%	30.0%	20.0%	0.0%	0.0%	0.0%	0.0%
Linbian	1231.17	19,235	100.0%	100.0%	100.0%	100.0%	55.2%	89.7%	96.6%	0.0%
Sinyuan	957.74	36,692	100.0%	100.0%	100.0%	100.0%	65.2%	30.3%	54.6%	0.0%
Wandan	906.33	52,085	100.0%	100.0%	96.6%	100.0%	5.7%	0.0%	0.0%	0.0%
Changzih	762.90	30,429	53.7%	43.3%	94.0%	70.2%	0.0%	0.0%	0.0%	0.0%
Linluo	695.76	11,313	100.0%	42.3%	100.0%	100.0%	0.0%	0.0%	0.0%	0.0%
Neipu	685.94	56,148	56.8%	45.5%	92.1%	23.9%	0.0%	0.0%	0.0%	0.0%
Jiadong	653.46	20,247	100.0%	100.0%	100.0%	100.0%	62.5%	35.4%	39.6%	0.0%
Dashu	644.81	43,190	98.2%	40.0%	96.4%	100.0%	0.0%	0.0%	0.0%	0.0%
Zhutian	609.46	17,719	100.0%	92.3%	57.7%	82.7%	0.0%	0.0%	0.0%	0.0%
Nanzhou	581.23	11,026	100.0%	100.0%	100.0%	100.0%	3.0%	48.5%	72.7%	0.0%
Jiouru	527.67	22,172	83.1%	49.4%	71.4%	84.4%	0.0%	0.0%	0.0%	0.0%
Kanding	523.70	16,374	97.6%	97.6%	90.5%	90.5%	7.1%	7.1%	33.3%	0.0%
Fangliao	441.36	25,482	98.7%	75.3%	98.7%	98.7%	42.9%	0.0%	0.0%	14.3%
Yanpu	413.82	26,629	27.9%	87.3%	26.6%	30.4%	0.0%	0.0%	0.0%	0.0%
Cishan	402.70	38,100	30.7%	59.1%	1.1%	11.4%	0.0%	0.0%	0.0%	0.0%
Ligang	389.06	26,814	52.1%	18.8%	7.3%	26.0%	0.0%	0.0%	0.0%	0.0%
Wanluan	344.43	20,918	55.7%	88.6%	67.1%	2.9%	0.0%	0.0%	0.0%	0.0%
Meinong	343.73	41,258	60.7%	77.4%	64.3%	29.8%	0.0%	0.0%	0.0%	0.0%
Gaoshu	283.08	25,520	5.5%	75.2%	61.5%	71.6%	0.0%	0.0%	0.0%	0.0%
Xinpi	173.39	10,232	98.8%	41.2%	83.5%	81.2%	0.0%	0.0%	2.4%	0.0%
Average	855.23	41,604.64	80.21%	75.12%	76.98%	72.38%	15.60%	13.53%	17.67%	0.57%
